# Supplementary figures and images for: The association of Helicobacter pylori infection with serum lipid profiles: An evaluation based on a combination of meta-analysis and a propensity score-based observational approach
Source: PLoS One. 2020 Jun 8;15(6):e0234433. doi: 10.1371/journal.pone.0234433 (PMC7279579; doi:10.1371/journal.pone.0234433)

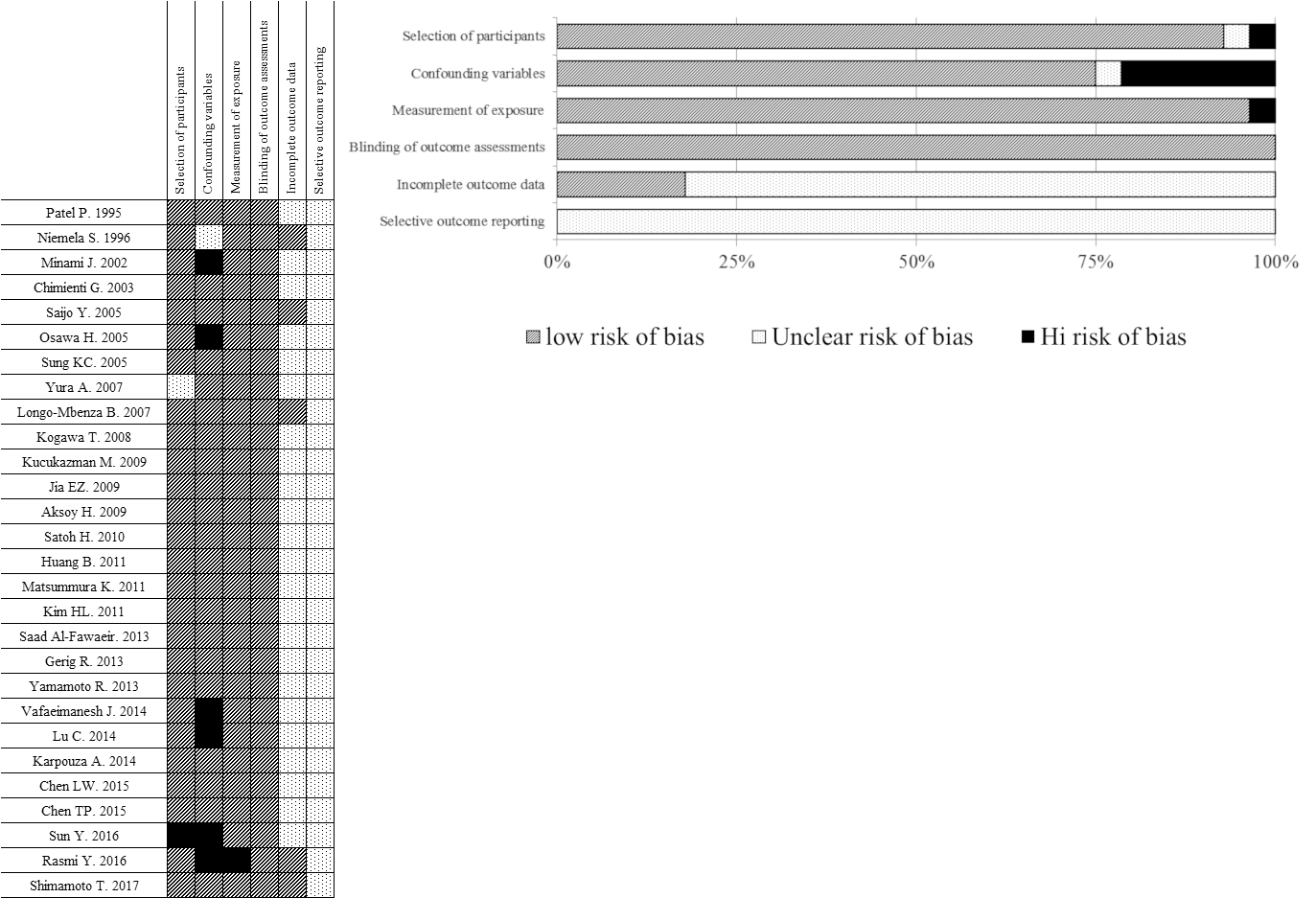

Supplement: S2 Fig — (A) The risk of bias summary by authors’ judgments about each bias element for each study. (B) The risk of bias graph by assessment of the risk of bias across studies. Bias is assessed as judgment (high, low, or unclear) for individual elements. The diagonal stripes show a low risk of bias, pin dot shows unclear risk of bias and black fill shows a high risk of bias. (TIFF) [file pone.0234433.s007.tiff]

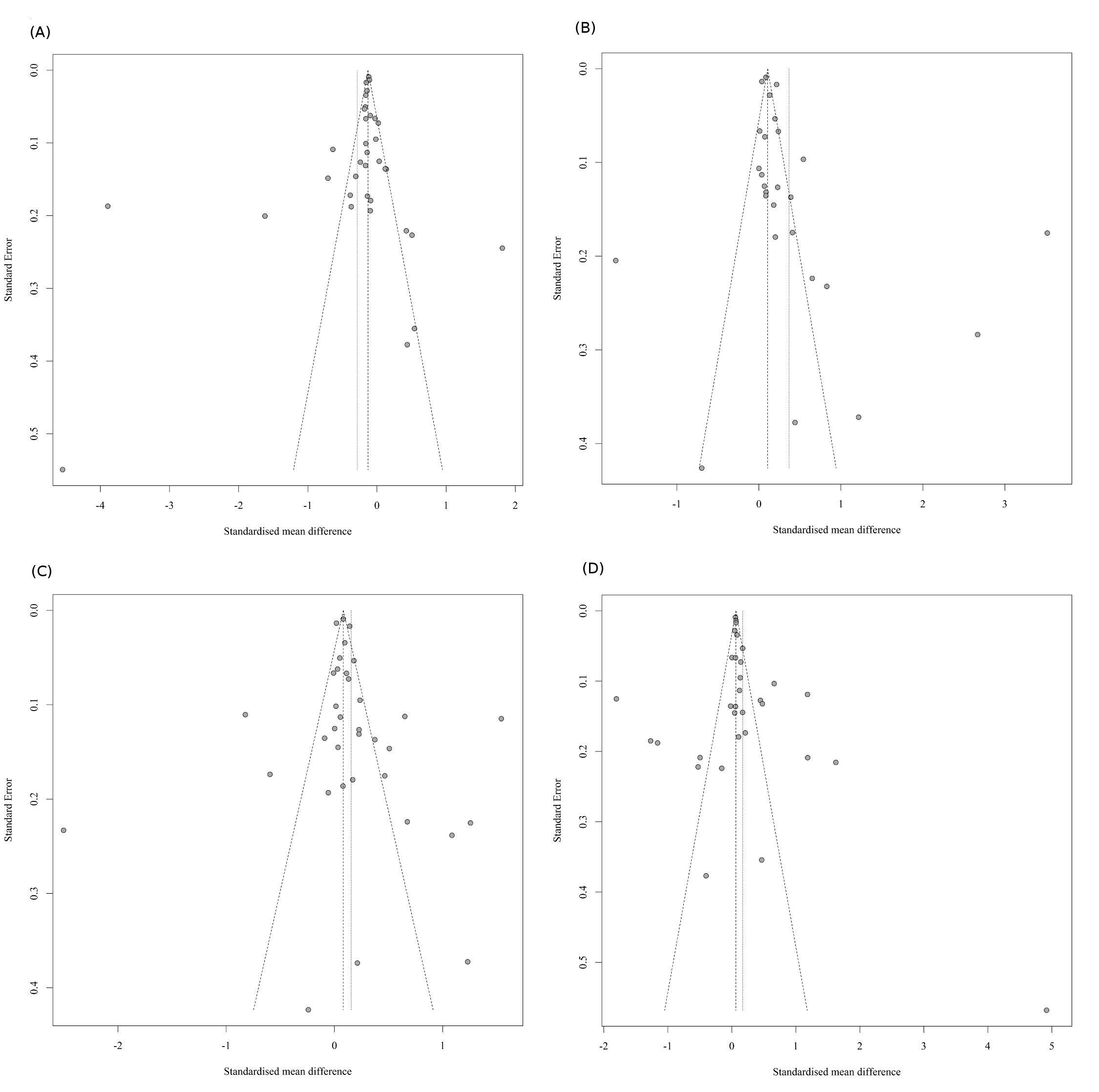

Supplement: S3 Fig — (TIFF) [file pone.0234433.s008.tiff]
